# Trauma-informed Temporary Assistance for Needy Families (TANF): A Randomized Controlled Trial with a Two-Generation Impact

**DOI:** 10.1007/s10826-017-0987-y

**Published:** 2018-01-01

**Authors:** Layla G. Booshehri, Jerome Dugan, Falguni Patel, Sandra Bloom, Mariana Chilton

**Affiliations:** 1 College of Nursing & Health Professions, Drexel University, 1601 Cherry Street, Philadelphia, PA 19102 USA; 2 Dornsife School of Public Health, Drexel University, 3600 Market Street, 7th Floor, Philadelphia, PA 19104 USA

**Keywords:** TANF, Randomized controlled trial, Two-generation, Depression, Trauma

## Abstract

Temporary Assistance for Needy Families (TANF) has limited success in building self-sufficiency, and rarely addresses exposure to trauma as a barrier to employment. The objective of the Building Wealth and Health Network randomized controlled trial was to test effectiveness of financial empowerment combined with trauma-informed peer support against standard TANF programming. Through the method of single-blind randomization we assigned 103 caregivers of children under age six into three groups: control (standard TANF programming), partial (28-weeks financial education), and full (same as partial with simultaneous 28-weeks of trauma-informed peer support). Participants completed baseline and follow-up surveys every 3 months over 15 months. Group response rates were equivalent throughout. With mixed effects analysis we compared post-program outcomes at months 9, 12, and 15 to baseline. We modeled the impact of amount of participation in group classes on participant outcomes. Despite high exposure to trauma and adversity results demonstrate that, compared to the other groups, caregivers in the full intervention reported improved self-efficacy and depressive symptoms, and reduced economic hardship. Unlike the intervention groups, the control group reported increased developmental risk among their children. Although the control group showed higher levels of employment, the full intervention group reported greater earnings. The partial intervention group showed little to no differences compared with the control group. We conclude that financial empowerment education with trauma-informed peer support is more effective than standard TANF programming at improving behavioral health, reducing hardship, and increasing income. Policymakers may consider adapting TANF to include trauma-informed programming.

## Introduction

The Temporary Assistance for Needy Families program (TANF) is meant to help low income caregivers gain employment skills, secure employment and reach self-sufficiency. However, after 20 years of research on the impacts of TANF, it is clear that it falls short of helping people enter the workforce and stay there, and that TANF participants have serious behavioral health challenges that affect their ability to reach self-sufficiency (Bryner and Martin 2005; Dworsky and Courtney 2007; Martin and Caminada 2011). In order to receive TANF, caregivers with young children under age six are required to participate in 20 “work hours” per week that may include job search, training, or other programming. However, evidence shows that the majority of such programs do not address the well-being of families, nor are there incentives to help caregivers find steady well-paying opportunities (Corcoran et al. 2004; Danziger 2010; Hildebrandt and Stevens 2009; Kaplan et al. 2005). In many instances, TANF participants may get jobs, but do not succeed in keeping them, only to return to TANF again (Hildebrandt and Kelber 2012; Hildebrandt and Stevens 2009; Ziliak 2014).

Almost one third of TANF recipients have a work-limiting health condition, and high rates of exposure to violence and adversity in their families and communities (Cheng 2013; Kennedy 2006; Lown et al. 2006). Adverse childhood experiences (ACEs) consisting of physical and emotional abuse and neglect, sexual abuse, and household dysfunction, such as having a household member in prison or witnessing domestic violence, are especially prevalent among those receiving TANF (Cambron et al. 2014). The original ACEs studies were conducted at Kaiser Permanente in Southern California in two waves of data collection of over 17,000 members of their Health Maintenance Organization, where adverse childhood experiences reported by survey respondents were compared with current health status and behaviors (Anda et al. 2006; Felitti et al. 1998). Since then additional research studies have linked ACEs to work-limiting conditions such as depression, cardiovascular disease, autoimmune diseases, and food insecurity, while damaging work prospects and stable income (Adams et al. 2013; Anda et al. 2008; Breiding et al. 2014; Cambron et al. 2015; Chilton et al. 2015; Danese et al. 2009; Dube et al. 2009; Staggs et al. 2007). High exposure to adversity among TANF-eligible caregivers also has crippling effects for academic achievement, parenting, employment, and executive functioning capabilities (Evans et al. 2011; Liu et al. 2013; Lu et al. 2008; Randles 2014). As an antidote, building up social support and promoting resilience have been shown to help interrupt the cycle of adversity (Kneipp et al. 2011; Larkin et al. 2014; Vayshenker et al. 2016). In addition, trauma-informed approaches that integrate knowledge and awareness of how trauma affects cognitive, social and emotional functioning, that seek to ensure that operations and processes do not re-traumatize individuals and incorporate group therapy approaches, have shown promise for reducing depression and other trauma-related symptoms (Bethell et al. 2014; Staub and Vollhardt 2008). Based on this research, it is clear that trauma-informed approaches may benefit TANF programming that seeks to improve employment and self-sufficiency outcomes for low income caregivers, many of whom have experienced adverse childhood experiences, intimate partner violence and community violence.

Despite the growing evidence that ACEs and community violence are prevalent among low income caregivers (Anda et al. 2006; Baglivio et al. 2014; Dreier et al. 2001; Peterson and Krivo 2005), TANF is one of the public assistance programs that has seen many attempts to improve outcomes without attention to this growing scientific base. Numerous randomized controlled trials have sought to improve employment outcomes, and these interventions have met with varied success (Falk 2012; Gueron and Rolston 2013). The majority of these large trials have integrated approaches that work with each client on an individual basis to connect them with employment and/or education, and to reduce participation in TANF (Gueron and Rolston 2013). Other TANF RCT’s have sought to address health barriers through referrals and home visits (Kneipp et al. 2011, 2013). To date, however, there has been no intervention that has sought to integrate trauma-informed practice and programming into TANF programming for caregivers who are required to carry out work participation in order to receive benefits. In addition, there is growing interest in how public assistance programs affect both caregiver and child. Recognizing that childhood experiences shape adult behavior, health and income, and, in turn, that caregivers’ health and success shape the health and wellbeing of their young children, government agencies have begun to adopt a two-generation framework that integrates some attention to child health (Chase-Lansdale and Brooks-Gunn 2014; Office of Family Assistance PeerTA Network 2015; Shonkoff and Fisher 2013).

This randomized controlled trial, The Building Wealth and Health Network (The Network RCT), sought to reduce economic hardship and behavioral health challenges associated with self-sufficiency among families with at least one child under age 6 who were required to fulfill 20 h of work participation each week. The analysis examines the impact of The Network RCT 28-week curriculum on behavioral health outcomes, economic hardship, and labor force participation among TANF participants by investigating three questions. Our first aim was to identify if there was selection bias in follow up response rates that could lead to erroneous differences in outcome measurements unrelated to treatment assignment. Secondly, we hypothesized that compared to the control group, intervention participants would experience statistically significant improvements in behavioral health, economic hardship, and labor market outcomes after exposure to the intervention. Thirdly, we hypothesized that compared to those that had low participation in the intervention, those that had greater exposure to the interventions would report improvements in health, hardship, and employment.

## Method

### Participants

Participants were primary caregivers of young children under the age of six who were receiving temporary assistance for needy families and who are required to work at least 20 h per week in order to receive these benefits. Recruited by research staff at three county assistance offices, the 103 participants were randomized into three groups: 31 in the control group, 35 in the partial intervention, and 37 in the full intervention (Table 1). While basic characteristics have already been reported in a previous publication (Sun et al. 2016), we highlight across all groups high rates of depressive symptoms ranging from 49 to 62%, and at least one concern of child developmental risk ranging from 12.9 to 22.9%, and over half of the participants reporting moderate to severe food, housing, or utility hardship. Additionally, over 90% of the sample was unemployed at baseline.

**Table 1:** Baseline characteristics of participants in the Building Wealth and Health Network RCT, Philadelphia 2014–2015

	Intervention groups					
	Control (*n* = 31)		Partial (*n* = 35)		Full (*n* = 37)	
	Mean	SD	Mean	SD	Mean	SD
Child’s age (months)	30.9	(16.0)	29.1	(17.8)	31.3	(21.5)
Caregiver’s age	26.4	(4.3)	24.6	(5.6)	25.3	(5.5)
	N	%	N	%	N	%
Caregiver gender						
Female	30	(96.8)	32	(91.4)	35	(94.6)
Male	1	(3.2)	3	(8.6)	2	(5.4)
Immigration status						
US Born	31	(100.0)	34	(97.1)	36	(97.3)
Foreign born	0	0	1	(2.9)	1	(2.7)
Race/ethnicity						
Black non-Hispanic	25	(80.6)	30	(85.7)	36	(97.3)
Hispanic	3	(9.7)	1	(2.9)	1	(2.7)
Other	3	(9.7)	2	(5.7)	0	0
White non-Hispanic	0	0	2	(5.7)	0	0
Sexual orientation						
Heterosexual	24	(77.4)	29	(82.9)	33	(89.2)
Bisexual	6	(19.4)	4	(11.4)	4	(10.8)
Gay or lesbian	1	(3.2)	2	(5.7)	0	0
Marital status						
Living with a partner	4	(12.9)	5	(14.3)	3	(8.1)
Married	0	0	0	0	1	(2.7)
Never married	27	(87.1)	29	(82.9)	31	(83.8)
Separated	0	0	1	(2.9)	2	(5.4)
Education						
Some high school or grade school	7	(22.6)	11	(31.4)	12	(32.4)
High school grad or GED	11	(35.5)	14	(40.0)	10	(27.0)
At least some college and above	13	(41.9)	10	(28.6)	15	(40.5)

Chi-square and Wilcoxen-Mann Whitney tests analysis showed no between-group differences, except for caregiver age (*p* = 0.07). See (Sun et al. 2016) for more comprehensive review

### Procedure

Through single-blind randomization participants were assigned into each group. After consenting to participate, participants completed baseline and follow-up surveys every 3 months over 15 months and received additional resources and the opportunity to speak with a social worker if needed. We conducted a mixed effects analysis to compare baseline to post-program outcomes at months 9, 12, and 15. In a separate analysis, we included class participation as a control to model impact of class participation on outcomes for the partial and full groups. While this does not necessarily indicate adherence (Fixsen et al. 2005), class participation is an indication of amount of exposure to the group process so essential to learning about finances, sharing resources, and having opportunities to build self-efficacy. Recruitment and randomization processes were successful, as there were no statistically significant differences in all characteristics and baseline outcomes by group. Baseline outcomes from our sample show high rates of exposure to ACEs and community violence. Almost 40% of all caregivers reported experiences of four or more adversities in their childhood, including abuse, neglect and household dysfunction; 64.7% of caregivers had seen a seriously wounded person after an incident of violence, and 27.2% had seen someone killed. Caregivers reported on the health and wellbeing of their children at baseline, and 36% reported their young children to be at risk for cognitive, social, and emotional delay, and almost half (48.5%) of their fathers spent time in prison. A full description of methods and baseline characteristics are outlined in a previous publication (Sun et al. 2016). The Network RCT ran from June 2014 to December 2015; Clinical trial registration number NCT02577705.

We used Audio Computer-Assisted Self-Interview (ACASI) software to administer all surveys regarding demographics, economic hardship, behavioral health, exposure to adversity and violence, and labor market outcomes. The research staff included measures validated by the clinical literature within the survey to ensure the external validity of the findings and tested glitches and readability with multiple respondents who are similar to those in the study. On average, the baseline survey took about 60 min to complete; each follow-up survey took approximately 30 min. Each participant was compensated $25 dollars for participating in each survey. With six total surveys, participants had the opportunity to receive up to $150. Responses for follow-up questionnaires in months 3 and 6 were excluded from the study sample, as they were administered before the end of the 28-week curriculum.

The Network RCT included three groups: control, a partial intervention and full intervention. The control group received standard TANF programming consisting of 20 h per week of scheduled supervised job training and job search activities. The partial intervention group received assistance in opening a credit union savings account, into which their own savings were matched by The Network RCT. It also included 28-weeks of financial empowerment education in weekly 3-hour classes. Content focused on identifying and harnessing internal and external resources to take steps towards self-sufficiency with education that included basic concepts of saving for education, housing, entrepreneurial activities, and retirement, and improving credit and reducing debt. The full intervention group received the same financial empowerment education and matched savings accounts as the partial group, with an added 28-week 4-hour peer support group called Self-Empowerment Groups. The group name drew from the Sanctuary Model®, a trauma-informed approach to social services (Bloom and Sreedhar 2008). The curriculum drew on key components from the model’s S.E.L.F. framework by focusing on four domains: creating physical, psychological, social and moral safety (S), processing and managing emotions (E), recognizing loss and letting go (L), and developing goals for a sense of future (F). The language of S.E.L.F. establishes a common framework that helps people who have experienced adversity to work towards building a stable foundation that supports their relationships with each other, within their families and communities, and gives opportunities for people to express their goals and potential for success. Financial empowerment classes were led by a facilitator contracted through a local financial services organization. S.E.L.F. groups were led by two trained peer group facilitators.

### Measures

This study examines measures of family behavioral health (depression, self-efficacy, and child developmental risk), economic hardship (hardship index), and labor market outcomes (employment status, earnings).

Depressive symptoms were measured using the validated 10-item Center for Epidemiologic Studies Depression Scale (CES-D)(Kohout et al. 1993; Radloff 1977). Which is reliable and consistent with the original version across a wide variety of populations (Hann et al. 1999; Zhang et al. 2012). Each item measures depressive symptoms on a 3-point scale (30 points total). Higher scores reflect greater depressive symptoms; a score of 10 or more is an indication of clinical depression.

Ability to manage stress, and capacity to address challenges is measured using the 10-item General Self-Efficacy Scale (GSE) which has strong validity and reliability across numerous populations including low income caregivers (Scholz et al. 2002; Schwarzer and Jerusalem 1995). Each item represents a measurement of an individual’s ability to deal with different demanding situations on a 4-point scale (38 points total); higher scores reflect a participant’s greater ability to deal with demanding situations.

Child’s developmental risks were measured using the 10-item Parent’s Evaluation of Developmental Status Scale (PEDS) (Glascoe 1998b). Each item measures developmental risk on a 3-point scale; only affirmative responses to developmental risk questions based on child’s age are tallied (10 points total). These data are used to construct a developmental risk indicator, equal to 1 if one or more developmental risks are reported, and 0 otherwise. PEDS has been validated with many disadvantaged US populations, and has a sensitivity of 91–97% and specificity of 73–86% (Glascoe 1998c). One or more developmental risks reported by the parent, are associated with significant disability in adult life (Glascoe 1998a, 2003; Glascoe and Marks 2011).

We measured economic hardship with an index that aggregates responses from three validated measures: the U.S. Household Food Security Survey Module (HFSSM), an energy security survey, and housing security survey. Each construct generates 3 mutually exclusive categories to capture levels of material hardship in the previous 3 months. The HFSSM is a validated 18-item scale developed by U.S. Department of Agriculture to measure household food insecurity, meaning the lack of access to enough food for an active and healthy life for the household and/or children (Bickel et al. 2000), which as excellent reliability ranging from 0.86 to 0.93 (Carlson et al. 1999). Households were coded as food secure, low food secure, very low food secure. Because food insecurity is related to other forms of hardship, we combined food insecurity with energy and housing insecurity based on previous research (Frank et al. 2010). Energy insecurity was coded as energy secure (no threatened or actual utility disconnections, no unheated/uncooled days, and no use of a cooking stove for heating), moderate energy insecurity (threatened utility disconnection because of nonpayment), or severe energy insecurity (unheated or uncooled day because of nonpayment, actual utility disconnection, and/or heating the residence with a cooking stove). Housing insecurity was categorized as housing secure (≤1 move in previous year and not crowded or doubled up), moderate housing insecurity (household is crowded and/or doubled up and has ≤1 move), or severe housing insecurity (household is crowded and/or doubled up and has moved ≥2 times). Crowding was defined as >2 people per bedroom and doubling up as a positive answer to the following question, adapted from the US Census: “Are you temporarily living with other people even for a little while because of economic difficulties?” (Cutts et al. 2011). Cumulative hardship index scores ranged from 0 to 6, with food, housing, and energy each contributing a possible score of 0 (secure), 1 (moderately insecure), or 2 (severely insecure) to generate scores indicating no hardship (score of 0 = 0), moderate hardship (scores of 1–3 = 1), or severe hardship (scores of 4–6 = 2).

Labor market outcomes include self-reported current employment status and hourly earnings. In regression analysis, hourly earnings are transformed into logs to address skewness.

### Data Analyses

Descriptive statistics summarize respondent characteristics and outcomes across intervention groups at baseline and identify that across all follow-up periods there were equivalent response rates. We analyzed differences in response profiles between treatment groups using multivariate linear mixed effects modeling, with participant as a random effect and time of assessment (baseline and 9, 12, and 15 months) and treatment group indicators (control, partial, full) as fixed effects. Other control variables in the model include gender, race/ethnicity, educational attainment, exposure to adversity and violence, and the interaction between time of assessment and class completion. In a separate analysis of the partial and full intervention participants only, we included class attendance to measure effects of class participation on participant outcomes. Least squares means were calculated using the mixed effects analysis and differences are reported across time and groups.

We chose mixed effects models over generalized linear models (GLM) or generalized estimating equation models (GEE) not only for their ability to control for the fixed effects that influence these changes in outcomes, but also for their ability to model correlation between measurements of the same participant through the inclusion of a random effect (Gardiner et al. 2009). Further, GLM has the strength of generating consistent estimates of regression parameters in the presence of data missing at random and non-ignorable missing data, which avoids the need to utilize complete case data to generate consistent coefficient estimates (Ibrahim et al. 2005). As per convention for studies with small sample size, *p*-value < 0.10 was considered to indicate significant differences between subgroups.

## Results

Aside from caregiver age, where the partial intervention group was slightly younger than the other groups (*p* = 0.07), there were no statistically significant differences in participant demographics observed, suggesting successful randomization. We display response rates and basic characteristics of the study sample in Table 2 for survey participation from baseline and follow-up months 9, 12, and 15. Response rates were 50% at month 9 (*n* = 52), 50% at month 12 (*n* = 53), and 45% at month 15 (*n* = 46). We conducted a rank test of the independence of response rates across groups by follow-up months, and found no significant differences in the distribution of treatment assignment over time (*p* = 0.9253).

**Table 2:** Characteristics of participants in the Building Wealth and Health Network RCT, over course of study, Philadelphia 2014–2015

	Time periods							
	Baseline (*n* = 103)		Month 9 (*n* = 52)		Month 12 (*n* = 53)		Month 15 (*n* = 46)	
	N	%	N	%	N	%	N	%
Intervention group								
Control	31	(30.1)	19	(36.5)	17	(32.1)	14	(30.4)
Partial	35	(34.0)	15	(28.8)	18	(34.0)	15	(32.6)
Full	37	(35.9)	18	(34.6)	18	(34.0)	17	37.0
Caregiver gender								
Female	97	(94.2)	48	(92.3)	49	(92.5)	42	(91.3)
Male	6	(5.8)	4	(7.7)	4	(7.5)	4	(8.7)
Immigration status								
US Born	101	(98.1)	50	(96.2)	51	(96.2)	45	(97.8)
Foreign born	2	(1.9)	2	(3.8)	2	(3.8)	1	(2.2)
Race/ethnicity								
Black non-Hispanic	91	(88.3)	47	(90.4)	49	(92.5)	42	(91.3)
Hispanic	5	(4.9)	1	(1.9)	1	(1.9)	1	(2.2)
Other	5	(4.9)	2	(3.8)	2	(3.8)	2	(4.3)
White non-Hispanic	2	(1.9)	2	(3.8)	1	(1.9)	1	(2.2)
Sexual orientation								
Heterosexual	86	(83.5)	46	(88.5)	47	(88.7)	41	(89.1)
Bisexual	14	(13.6)	6	(11.5)	6	(11.3)	5	(10.9)
Gay or lesbian	3	(2.9)	0	0	0	0	0	0
Marital status								
Living with a partner	12	(11.7)	5	(9.6)	7	(13.2)	5	(10.9)
Married	1	(1.0)	3	(5.8)	2	(3.8)	2	(4.3)
Never married	87	(84.5)	42	(80.8)	43	(81.1)	37	(80.4)
Separated	3	(2.9)	2	(3.8)	1	(1.9)	2	(4.3)
Education								
Some high school or grade school	30	(29.1)	15	(28.8)	14	(26.4)	14	(30.4)
High school grad or GED	35	(34.0)	16	(30.8)	15	(28.3)	14	(30.4)
At least some college and above	38	(36.9)	21	(40.4)	24	(45.3)	18	(39.1)

The Cochran-Armitage and Jonckheere-Terpstra tests showed no differences in the distribution of characteristics across time

Behavioral health, hardship, and labor market outcomes are displayed in Table 3. Participants in the full intervention experienced statistically significant declines in depressive symptoms by month 15 compared to baseline (−1.13 points; *p* = 0.0640) and this decline is significantly lower compared to the control group at month 15 (*p* = 0.0154). Neither participants in the control group nor the partial intervention experienced any statistically significant changes in depressive symptoms. Compared to the baseline, the full intervention experienced an increase in self-efficacy at month 9 (1.08 points; *p* = 0.0388). During the same time period, the control group experienced a statistically significant decline in self-efficacy at month 9 (−2.84 points; *p* = 0.0589). Neither participants in the partial or full intervention experienced statistically significant changes in child developmental risks. However, among the control group, compared to the baseline, there was a statistically significant increase in the probability of reporting child development risks at month 9 (21%; *p* = 0.0680).

**Table 3:** Mixed effects analysis of behavioral health, economic hardship, and labor market outcome changes in the control, partial and full intervention groups in the Building Wealth and Health Network RCT, Philadelphia 2014–2015

Assessment period^a^								
Measure	Baseline	9 mo.	9 mo. vs Baseline	12 mo.	12 mo. vs Baseline	15 mo.	15 mo. vs Baseline	Group^b^
	LSM	LSM	*p-*value	LSM	*p-*value	LSM	*p-*value	*p*-value
Adult depressive symptoms								
Control group	10.66	9.55	0.3998	11.55	0.5134	12.83	0.1349	
Partial intervention	9.02	9.46	0.3689	9.97	0.9712	11.36	0.9215	0.4098
Full intervention	9.78	10.36	0.3085	10.26	0.8036	8.65	**0.0640**	**0.0154**
Adult self-efficacy								
Control group	31.89	29.05	**0.0589**	30.36	0.3212	31.10	0.6293	
Partial intervention	29.59	29.15	0.2237	30.89	0.1438	32.15	0.1064	0.5903
Full intervention	31.90	32.98	**0.0388**	32.39	0.2950	32.62	0.4537	0.4170
Child’s developmental risk								
Control group	0.10	0.31	**0.0680**	0.10	0.9507	0.09	0.8882	
Partial intervention	0.23	0.29	0.5741	0.14	0.4239	0.17	0.6031	0.5575
Full Intervention	0.11	0.19	0.4568	0.28	0.1321	0.27	0.1612	0.1883
Hardship index								
Control group	2.39	2.16	0.5590	2.60	0.6115	2.27	0.7868	
Partial intervention	2.42	2.12	0.4690	1.97	0.2457	2.04	0.3479	0.6663
Full intervention	2.59	2.56	0.9557	1.86	**0.0640**	2.19	0.3268	0.8783
Employment								
Control group	0.12	0.43	**0.0068**	0.61	**<0.0001**	0.38	**0.0384**	
Partial intervention	0.08	0.56	0.2511	0.57	0.9751	0.48	0.3508	0.4867
Full intervention	0.04	0.43	0.5951	0.44	0.5637	0.51	0.1570	0.3413
Earnings^c^								
Control group	2.40	2.26	0.5998	2.18	0.4307	2.45	0.8720	
Partial intervention	2.14	2.14	0.6773	2.18	0.4300	2.17	0.9578	**0.0793**
Full intervention	2.01	2.25	0.2471	2.37	**0.0857**	2.43	0.2853	0.8769

^a^ All values are least squares means. Statistically significant *p* values at *p* < 0.10 are shown in bold

^b^ Group difference at month 15. The excluded category is the control group

^c^ The mixed effects model was estimated in the log of earnings, but the least squares estimates of earnings are reported for readability. The *p* values reported in this section are from the log of earnings mixed effects analysis

Compared to baseline, participants in the full intervention experienced statistically significant declines in economic hardship by month 12 (−0.73 points, *p* = 0.0640). Neither the control nor partial intervention reported statistically significant changes in hardship throughout the study period.

The control group experienced statistically significant increases in employment in every follow-up period. In particular, employment increased by 26 percent (*p* = 0.0384) by month 15. Neither the partial nor full intervention reported significant changes in employment over the study period. However, compared to baseline, the full intervention experienced a statistically significant increase in earnings by month 12 (*p* = 0.0857), while the control and partial intervention groups reported no significant changes in hourly earnings.

The average class attendance for the partial and full intervention at the end of the 28-week education program was 26.0 and 23.6%, respectively (Table 4). This leads to an important question of whether increasing exposure to either intervention program could lead to increased positive impact on participant outcomes. Increased class participation was not associated with statistically significant changes in adult depressive symptoms, child development risk, self-efficacy, economic hardship, employment, or earnings for the partial intervention group. However, increased class attendance was associated with statistically significant improvements in some outcomes for the full intervention. In particular, the mixed effects coefficient estimates for class participation presented in Table 4 demonstrate that increasing class attendance by one percent was associated with decreases in developmental risks for the participant’s youngest child (coefficient estimate: −0.0048, *p* = 0.0284), non-significant increases in self-efficacy (coefficient estimate: 0.0463, *p* = 0.1048), and increased probability of employment (coefficient estimate: 0.0048, *p* = 0.0443).

**Table 4:** Mixed effects analysis of the impact of class attendance on behavioral health, economic hardship, and labor market outcomes in the Building Wealth and Health Network RCT, Philadelphia 2014–2015

	Dependent variables					
	Depressive symptoms		Development risk		Self-efficacy	
	Partial	Full	Partial	Full	Partial	Full
Attendance rate (%)^a^	−0.0526	−0.0276	−0.0001	−**0.0048**	0.0234	0.0463
	*P* = 0.1283	*P* = 0.3916	*P* = 0.9563	***P*** ** = 0.0284**	*P* = 0.5754	*P* = 0.1048

	Hardship index		Employment		Log of earnings	
	Partial	Full	Partial	Full	Partial	Full
Attendance rate (%)^a^	−0.0061	−0.0069	0.0030	**0.0048**	0.0041	−0.00204
	*P* = 0.2853	*P* = 0.2174	*P* = 0.2849	***P*** ** = 0.0443**	*P* = 0.2227	*P* = 0.3341

Significant *p* values (*p < *0.10) are shown in bold

^a^ At the end of the 28-week education program, the average attendance rate for the partial intervention group was 26.0% and for the full intervention group was 23.6%

## Discussion

Results demonstrate that the randomization was effective. Our survey response rate ranged from 45–50%, which is higher than average for at risk low-income caregivers (Western et al. 2016). There were no significant differences by group in terms of baseline and follow-up characteristics and survey response rate. This suggests that the results, where groups are compared both within and across groups, are likely due to the intervention itself.

The Network RCT intervention demonstrated important and diverse findings. Changes in health, economic hardship and employment varied at each follow-up time point. This is reflective overall that behavioral and economic changes do not happen simultaneously and that effects may change over time. The improvements for caregiver in self-efficacy and depression are promising, not only because they demonstrate improvements in emotional and behavioral wellness, but also because of their positive impacts on employment. The demonstrated improvements in self-efficacy by month 9 for the full intervention suggests that an underlying challenge in securing and maintaining employment can be addressed and that it may have positive impacts on employment. Self-efficacy is associated with greater motivation and job satisfaction, self-leadership strategies and job performance, which are all necessary for success in the work force (Cherian and Jacob 2013). Length of time in the full intervention was associated with improvements in self-efficacy, though these results were only significant at the 90% confidence level. For the control group, self-efficacy reduced at month 9, and stayed lower than the other groups in the partial and full intervention, and results suggest that the longer someone participated in the peer support group, the more likely self-efficacy improved. The ability of the program to reduce depressive symptoms was most effective for the full intervention group by month 15, suggesting that a shift in mental health takes a significant amount of time. Trauma-informed group therapy is known to have positive effects on behavioral health and parenting practices (Murphy et al. 2015). The Network RCT’s weekly sessions had a significant clinical impact suggesting profound health effects for non-medical, trauma-informed interventions. This is especially important because any type of reduced depression is known to have positive effects on helping individuals to secure and maintain employment (Schoenbaum et al. 2002). Both improved self-efficacy and reduced mental health are known to improve parenting practices, and therefore have an impact on the wellbeing of children (Kohlhoff and Barnett 2013).

This two-generation effect is reflected in The Network RCT results. Though this study worked with the adult caregiver and not the whole family through our programming, based on other research, it is likely that these demonstrated improvements in caregiver self-efficacy, depression, and economic security are linked to improved parenting, and hence the protection of well-being among their young children (Schmit et al. 2014; Shonkoff and Fisher 2013; The Annie E. Casey Foundation 2014; Administration for Children and Families Office of Family Assistance 2016). Evidence here is in child developmental risks. Caregivers in the intervention groups reported no changes in developmental risk concerns; however, the control group reported statistically significant increases in child developmental risks. Not only do increased self-efficacy and improvement in depression potentially buffer children from developmental risk, but increased risk in the control group is consistent with other studies that demonstrate that participation in regular TANF programming may be associated with poorer child development outcomes (Heflin and Acevedo 2011). Additionally, since the control group received no intervention to help mitigate depressive symptoms, this result is consistent with reports that caregiver depression is linked with heightened risk of developmental problems in young children, either through disrupted parenting practices, or in response to a child’s developmental delay (E. Cheng et al. 2015; Rose-Jacobs et al. 2008). When controlling for the extent to which participants are exposed to the intervention, the greater the participation in the full intervention, the *less* likely one was to report developmental risks for their children. Overall, results demonstrate that incorporating trauma-informed peer support into a TANF curriculum will likely have positive benefits on caregiver depression, self-efficacy and on child wellbeing; however, the timing and endurance of these changes beyond month 9 and 15 is uncertain.

There is some research demonstrating that financial management skills are related to food insecurity and other forms of hardship (Gundersen and Garasky 2012), yet there has been no financial management intervention to date that shows effects on reducing food insecurity and other types of hardship with basic needs. With the Network RCT, hardship with food, housing, and energy between the groups were significant. Statistically significant reductions in hardship were observed for the full intervention at month 12, but they were not observed in either the control or partial intervention groups. This suggests that although financial empowerment training may provide opportunities to build capabilities to address economic adversity and manage paying bills on time (or more consistently), the addition of trauma-informed care may help participants improve overall economic stability.

Labor market results were mixed. The control group saw greater increases in employment compared to the partial and full intervention groups. This is likely a result of standard TANF programming that has the singular focus on mandating that people find employment as quickly as possible. It is important to note, however, that there were no significant differences in reported income between the full intervention group and the control group, while the partial intervention group earnings remained lower than both groups throughout. These results demonstrate that while employment may improve, it may not substantially change a caregiver’s income. Indeed, the full intervention group showed a steady income increase over time, rather than income fluctuation that was reported in the control group. Finally, the amount of participation in the classes had little to do with income, but was associated with improvements in employment for the full intervention group.

The generalizability of our findings is limited by our analysis of TANF beneficiaries that reside in one city. However, since the means tests to determine TANF eligibility are similar across states, TANF populations will have similar demographic compositions across the United States (Kim and Fording 2010). Secondly, class participation rates were limited (though no different than participation in standard TANF programming in the state) and potentially hindered our ability to assess full treatment effects. Nevertheless, our sensitivity analysis suggests that even partial exposure to the full intervention has significant impacts on participants’ behavioral and economic outcomes. Though there was noticeable survey attrition across time that may impact the precision of our estimates (Crosby et al. 2010), there were still no significant differences by group across time suggesting successful retention strategies and internal validity to our results (Brannon et al. 2013; Yancey et al. 2006). Finally, all data are self-reported, and therefore may be prone to bias, where people may minimize symptoms or other concerns and exaggerate income and employment experiences.

Overall, our results, while promising, suggest the need for a re-design of the intervention for full implementation beyond the research phase. Promising areas are in the integration of financial empowerment education into the curriculum of the trauma-informed peer support. As well, attrition over time, suggests that the curriculum should be shortened to demand less overall time of families that are already struggling to find time for self-care so that participation in the class can be more robust.

Results of the Network RCT suggest that trauma-informed approaches may create steady improvements in health and income. Future research should explore how such approaches may help participants improve income and wellbeing over a longer period of time for caregiver and child.
